# Response to: Comment on “Assessment of Liver Stiffness in Pediatric Fontan Patients Using Transient Elastography”

**DOI:** 10.1155/2017/4217878

**Published:** 2017-02-08

**Authors:** Becky Chen, Richard A. Schreiber, Derek G. Human, James E. Potts, Orlee R. Guttman

**Affiliations:** ^1^Department of Pediatrics, Children's Hospital of Western Ontario, London, ON, Canada; ^2^Division of Gastroenterology, Hepatology and Nutrition, British Columbia Children's Hospital, Vancouver, BC, Canada; ^3^University of British Columbia, Vancouver, BC, Canada; ^4^Children's Heart Centre, British Columbia Children's Hospital, Vancouver, BC, Canada

We thank Drs. Xue and Cai for their interest in our study and we appreciate their comments regarding its design [1, 2]. They identify an important factor in the interpretation of transient elastography (TE) results, namely, the potential for falsely elevated values in the context of raised alanine aminotransferase (ALT).

As referenced in our paper, recent data suggest that TE values may be 1.3–3 times higher in the setting of acute liver inflammation and moderately elevated ALT [3]. In the 2012 study by Tapper et al., increased levels of ALT correlated with liver stiffness among hepatitis C patients with METAVIR scores 0–2. A recent study of pediatric patients with a variety of liver diseases similarly found that the correlation between ALT and TE result was stronger among those with inflammatory diagnoses and F0/F1 fibrosis [4].

The patients included in the studies referenced by Drs. Xue and Cai all had acute or chronic hepatitis from inflammatory conditions, namely, infectious (HAV, HBV, and HCV), toxic, or autoimmune diagnoses. This is in important contrast to our cohort of Fontan patients. Histologic studies of liver biopsies after Fontan demonstrate varying degrees of portal and sinusoidal fibrosis, but inflammatory changes are quite rare [5, 6].

In our study, although the ALT levels among Fontan patients were significantly higher than those in the healthy controls, the absolute levels of ALT were within the normal reference ranges provided by the respective laboratories (12.0–54.0 U/L). This is also in contrast to the above-mentioned studies, where a majority of patients had ALT values that were elevated, in some cases considerably so.

In summary, while it is important to interpret TE results thoughtfully in the context of hepatic inflammation and ALT elevation, we feel that this factor likely does not significantly contribute to the elevated TE values among our Fontan patients. TE values in our Fontan cohort likely represent the contributions of hepatic congestion and possible fibrosis. We agree, however, that further prospective studies involving TE are necessary to determine its optimal utility in this patient population.
